# Exceptionally preserved early Cambrian bilaterian developmental stages from Mongolia

**DOI:** 10.1038/s41467-021-21264-7

**Published:** 2021-02-15

**Authors:** Michael Steiner, Ben Yang, Simon Hohl, Da Li, Philip Donoghue

**Affiliations:** 1 College of Earth Science and Engineering, Shandong University of Science and Technology, Qingdao, China; 2 Department of Earth Sciences, Freie Universität Berlin, Berlin, Germany; 3 MNR Key Laboratory of Stratigraphy and Palaeontology, Institute of Geology, Chinese Academy of Geological Sciences, Beijing, China; 4 State Key Laboratory of Marine Geology, School of Ocean and Earth Sciences, Tongji University, Shanghai, China; 5 School of Marine Science and Engineering, Nanjing Normal University, Nanjing, China; 6 School of Earth Sciences, University of Bristol, Bristol, UK

**Keywords:** Evolutionary developmental biology, Palaeontology

## Abstract

Fossilized invertebrate embryonic and later developmental stages are rare and restricted largely to the Ediacaran-Cambrian, providing direct insight into development during the emergence of animal bodyplans. Here we report a new assemblage of eggs, embryos and bilaterian post-embryonic developmental stages from the early Cambrian Salanygol Formation of Dzhabkan Microcontinent of Mongolia. The post-embryonic developmental stages of the bilaterian are preserved with cellular fidelity, possessing a series of bilaterally arranged ridges that compare to co-occurring camenellan sclerites in which the initial growth stages retain the cellular morphology of modified juveniles. In this work we identify these fossils as early post-embryonic developmental stages of camenellans, an early clade of stem-brachiopods, known previously only from isolated sclerites. This interpretation corroborates previous reconstructions of camenellan scleritomes with sclerites arranged in medial and peripheral concentric zones. It further supports the conjecture that molluscs and brachiopods are descended from an ancestral vermiform and slug-like bodyplan.

## Abstract (web summary)

The Cambrian is known as a period of rapid animal diversification, but the development of these animals is not well characterized. Here, Steiner et al. describe a new assemblage of Cambrian eggs, embryos and early postembryonic stages from Mongolia that provides insight into ancient bilaterian development and evolution.

## Introduction

The origin and early evolution of animals is among the most formative episodes in Earth history, establishing the basis of modern animal biodiversity and, through their ecological expansion, precipitating a fundamental change to the Earth system^1^. Animal bodyplans emerged as a consequence of evolutionary changes in the genetic regulation of embryological development. Insights into developmental evolution are usually sought in comparative developmental biology but, surprisingly, the fossil record has begun to provide more direct insights, manifest as fossilized eggs, embryos, and larvae of marine invertebrates^2,3^. Existing reports include almost complete cnidarian life cycles^2,4,5^, a possible ctenophore embryo^6^, scalidophoran embryos, and larvae^7–10^, as well as possible mollusc embryos^4^ which have impacted on macroevolutionary theories of developmental model, life history strategy, as well as the evolution of bodyplan symmetry^3^. The influence of this dimension of the fossil record is strictly limited by a paucity of sites where they are preserved. So far, early developmental stages of invertebrates in Orsten-type preservation have been reported from the Ediacaran to Ordovician of South China, Australia, Laurentia, and Siberia^2–4,7,11–15^.

Here, we report a new assemblage from the Cambrian Stage 3 of the Mongolian Dzhabkan Microcontinent, that includes fossil eggs, embryos attributable to the early scalidophoran *Markuelia* and, in particular, microscopic bilaterian postembryonic developmental stages preserved to an almost unparalleled cellular fidelity. The juveniles may represent early stages in the life cycle of co-occurring tommotiids, such as *Camenella mongolica* and *Camenella parilobata*, in which case they would appear to corroborate the hypothesis of slug-like bodyplan for stem-brachiopods^16–23^ and, possibly, ancestral trochozoans.

## Results

### An exceptional new Orsten-type fossil deposit of Mongolia

**Fig. 1:**
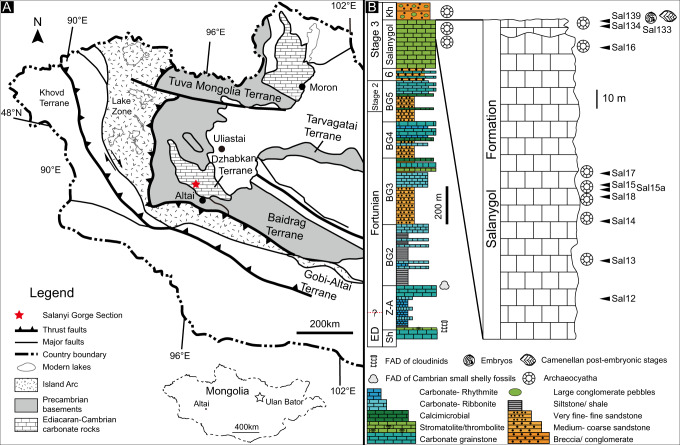
Geographic and stratigraphic occurrence of new assemblage of embryonic and postembryonic developmental stages. **A** Locality of Salanyi Gol (Gobi-Altai Province) on Dzhabkan Terrain of Mongolia. **B** Stratigraphic column (modified from ref. ^25^) with the distribution of the embryonic and postembryonic developmental stages. ED Ediacaran, Sh Shurgat Formation, Z-A Zuun Arz Formation, BG Bayan Gol Formation (numbers refer to members according^25^), Kh Khirekhan Formation, FAD first appearance datum; sample numbers: Sal 139 etc.

**Fig. 2:**
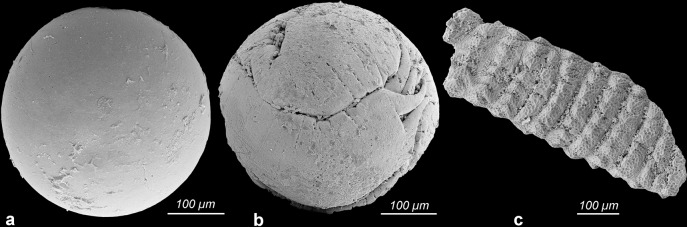
Phosphatized soft-bodied and cuticularized eggs, embryos, and articulated postembryonic developmental stages of the new Orsten-type fossil lagerstätte Salanyi Gol (Mongolia). **a** Egg. **b** Late-stage embryo of *Markuelia* with six large posterior hooks. **c** Articulated, hitherto undescribed slug-like organism with rows of cuticularized humps with net-like ornaments. All images are electron microscope micrographs. Specimen identifiers: **a** Sal 134-38; **b** Sal 134-16; **c** Sal 134-28.

**Fig. 3:**
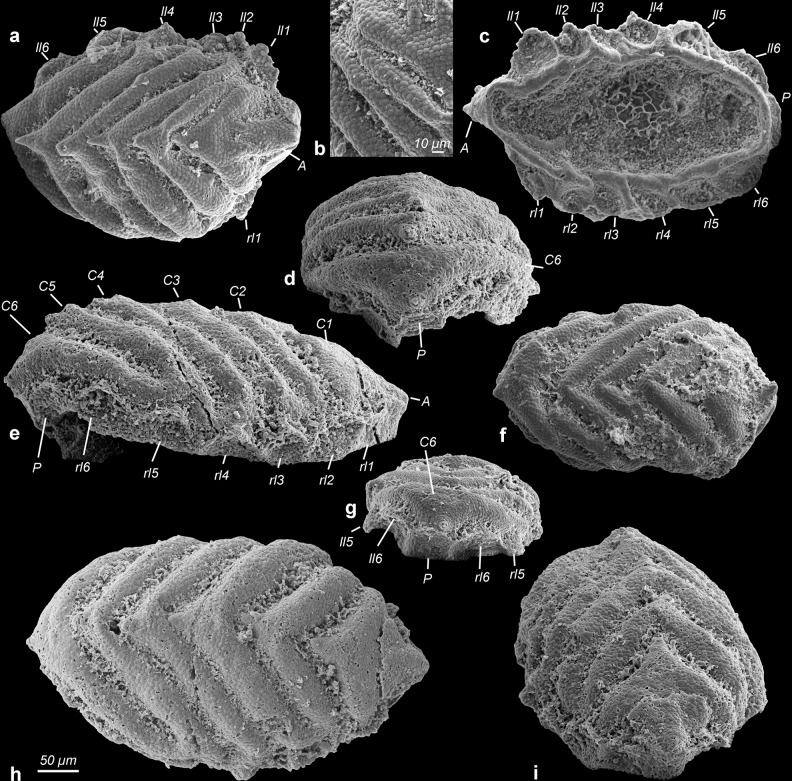
Camenellan juveniles from the Cambrian Salanygol Formation of Mongolia. **a** Dorsal view of an early juvenile with six arc-shaped ridges preserving cellular pattern; anterior nascent sclerite with different morphology. **b** Close-up of central ridges C1–3 with cellular pattern, enlargement of individual shown in **a**. **c** Ventral view of the early juvenile shown in **a**, with six pairs of lateral protuberances. **d** Posterior view, posterior nascent sclerite forms a small field only in this ontogenetic stage. **e** Lateral view on right peripheral zone with six small lateral protuberances. **f** Dorsal view of an individual with shallow central ridges. **g** Posterior view, note left lateral nascent sclerite five forms an asymmetric cone. **h** Dorsal view of an early juvenile with six arc-shaped central ridges. **i** Anterior view of **h** showing anterior nascent sclerite developing a different morphology than central nascent sclerites 1–6. C1–6 central nascent sclerite 1–6 of medial zone, P posterior nascent sclerite, A anterior nascent sclerite, ll1–6 left lateral sclerites 1–6 of peripheral zone, rl1–6 right lateral nascent sclerites 1–6 of peripheral zone. All images are electron microscope micrographs, all images at same scale except for **b**. Specimen identifiers: **a**–**d** Sal 139-019; **e**, **h**, **i** Sal 133-029; **f**, **g** Sal 133-43.

**Fig. 4:**
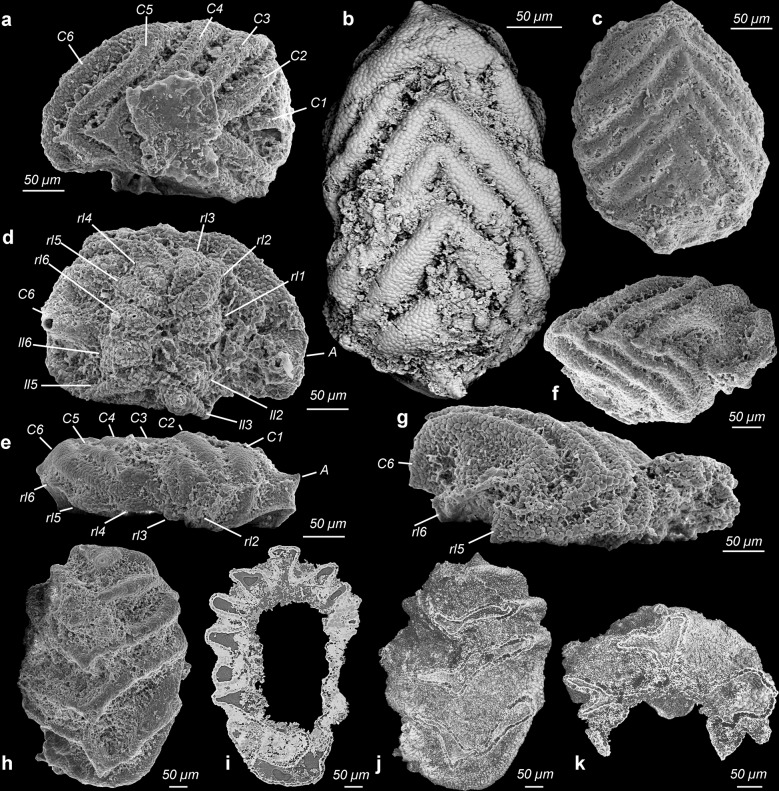
Camenellan juveniles from the Cambrian Salanygol Formation of Mongolia. **a** Dorsal view of an early juvenile with six central ridges preserving cellular pattern. **b** Dorsal view of the juvenile stage with six arc-shaped central ridges and one differently shaped anterior spinose structure. **c** Dorsal view of a juvenile with six shallow central ridges and an anterior field with a different morphology. **d** Ventral view of juvenile stage illustrated in **a**; 12 distinct fields form a band-like array in this ontogenetic stage, which later develop into the peripheral zone; note the circular plate-like anterior nascent sclerite with a small projection is outside the ventral band of fields; a posterior nascent sclerite is not yet developed or detectable. **e** Lateral view of an individual shown in **b** with shallow central ridges and the anterior nascent sclerite visible as an erect broad spine, lateral nascent sclerites are still not yet well developed, forming small fields at the bases of central ridges. **f** Slightly distorted and incomplete early juvenile with well-preserved cellular pattern. **g** Lateral view of incomplete early juvenile illustrated in **f**, where lateral protuberances begin to develop as asymmetric cones. **h** Larger juvenile with central ridges beginning to develop more erect structures. **i**, **j** Half volumes at different dorsoventral cutting planes of syncCT-models of individual shown in **h**, note single epithelial cell layer in sectioning plane. **k** Half volume of syncCT-model of **h** with cutting plan in anterior–posterior direction, revealing similar cellular pattern on the ventral side as visible dorsally. C1–6 central nascent sclerite 1–6 of medial zone, P posterior nascent sclerite, A anterior nascent sclerite, ll1–6 left lateral sclerites 1–6 of peripheral zone, rl1–6 right lateral nascent sclerites 1–6 of peripheral zone. All images are secondary electron micrographs of an electron microscope, except for **b**, which is a backscatter electron micrograph and **i**–**k**, which are volume models of X-ray syncCT. Specimen identifiers: **a**, **d** Sal 133-040; **b**, **e** Sal 133-43, **c** Sal 139-012; **g**, **f** Sal 133-042; **h**–**k** Sal 133-044.

The new assemblage of phosphatized eggs, embryos, and juveniles from Mongolia was discovered in the topmost phospholithoclastic carbonates of the Salanygol Formation at Salanyi Gorge (GPS N46° 48′ 32.1″ E095° 46′ 18.8″) located at the Dzhabkan Microcontinent (Fig. 1). The Ediacaran-early Cambrian transition sequence exposed in the Salanyi Gorge of Khasagt Khirekhan Mountain Range (Altai Province) is >1500 m thick^24,25^. This outcrop of the Terreneuvian Bayan Gol Formation exposes a >1140 m sequence of thick siliciclastics interlayered with thinner limestones or calcareous bioherms, overlain by massive limestones of the Salanygol Formation (ca. 360 m thick). The upper 160 m of the Salanygol Formation commonly contains redeposited archaeocyathans, while the lower part is a monotonous algal limestone devoid of a fossil fauna. Three thin horizons within the topmost 5 m of the Salanygol Formation contain phosphatized eggs, embryos, and postembryonic articulated life stages (Figs. 2–4), co-occurring with small shelly fossils (SSFs), such as *Lapworthella tortuosa, C. mongolica, C. parilobata, Salanygolina obliqua, Khairkhania rotata, Latouchella gobiica, Obtusoconus brevis, Yochelsonella crassa*, chancelloriids, and phospatized archaeocyathan fragments. The new deposit of phosphatized Orsten-type fossils is the first reported from the diverse Mongolian terrains of the Central Asian Orogeny Belt (CAOB). This fossil deposit is exceptional for preserving eggs, embryos, and cuticularized articulated organisms (Fig. 2). However, it is unusual for this type of fossil lagerstätte in preserving the cellular structure of tissues (Fig. 4) without extracellular cuticle or fertilization membranes.

The age of the Salanygol Formation has been the subject of some controversy^25,26^, alternately interpreted as Tommotian based on chemostratigraphy and a general lack of trilobites^25^, or Atdabanian–Botoman based on archaeocyathan and SSF assemblages^26–28^. The negative carbon isotopic shift in the lower Salanygol Formation coincides with a transition from boundstones with a higher amount of microbially derived fabrics to limestones with a higher portion of transported archaeocyathan bioclasts^29^. This may suggest that the carbon isotope shift in the Salanygol Formation does not reflect a global open seawater signature but, rather, a regional change in aquafacies, making it less informative stratigraphically. The most reliable age determination for the Salanygol Formation is therefore based on the occurrence of the archaeocyathans *Gordonicyathus howelli* and *Alataucyathus jaroschevitschi*^28^ both of which are typical for assemblages of the Altai-Sayan folded region of the CAOB, and correlate to the late Atdabanian of the Siberian Regional stratigraphic subdivision^30^. The Salanygol Formation is overlain unconformably by thick sandstones and conglomerates of the Khayrkhan Formation, correlating with the Botoman/Toyonian^28^.

### Bilaterian developmental stages preserved with cellular fidelity from Mongolia

**Fig. 5:**
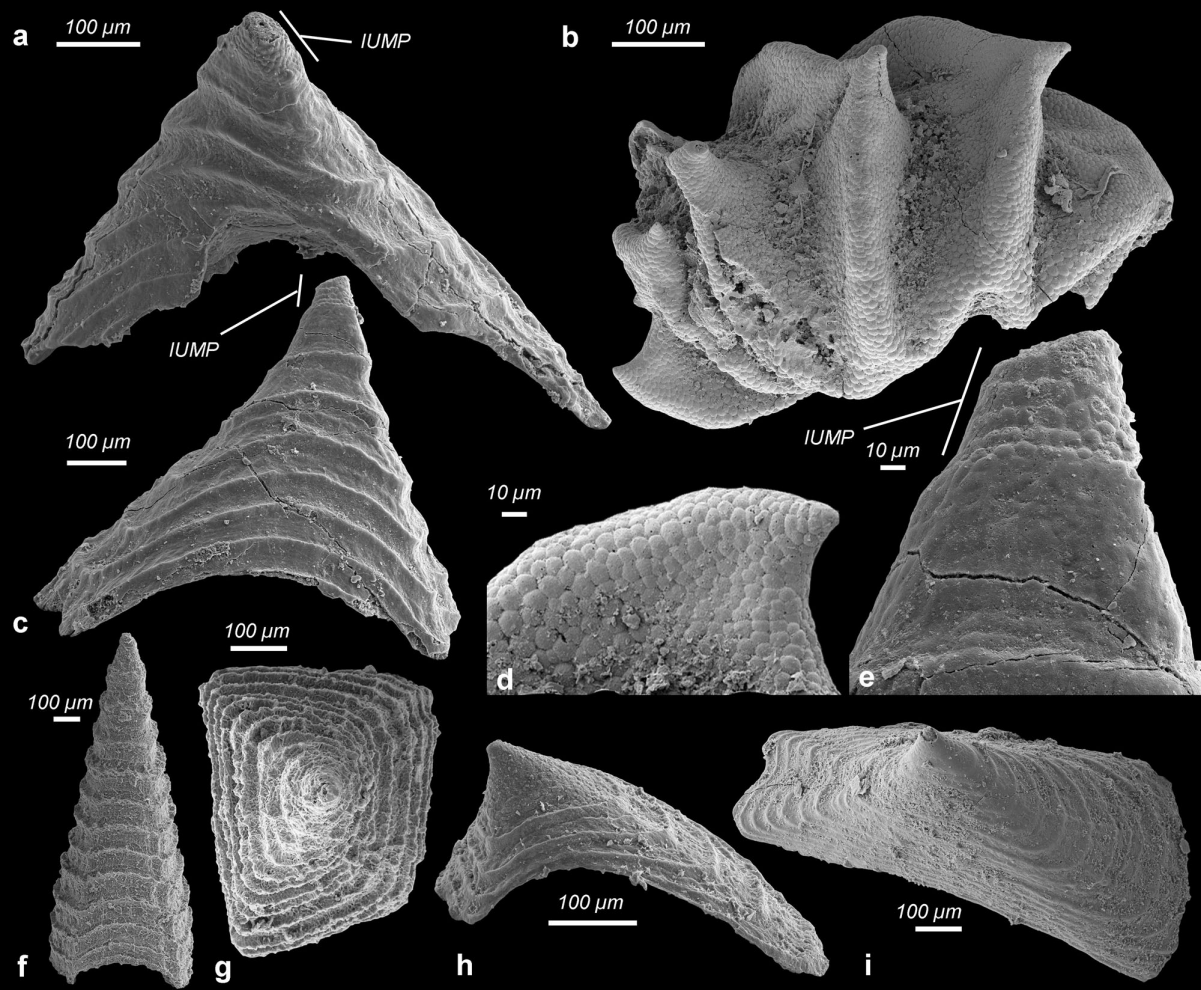
Camenellan juvenile and disarticulated tommotiid sclerites from the Cambrian Salanygol Formation of Mongolia. **a**, **c** Anterior and posterior views of disarticulated saddle-shaped sclerites of *C. mongolica* co-occurring with camenellan juvenile; note the partly broken tip preserves cellular pattern only visible in the juvenile part, but not in biomineralized parts with co-marginal ribs. **b** Dorsal view of an incomplete camenellan juvenile with six central erect spinose nascent sclerites; note cellular pattern still preserved on whole dorsum. **d** Detail of cellular pattern in a central nascent sclerite C1 of camenellan juvenile prior to onset of biomineralization, enlargement from **b**. **e** Detail of cellular pattern in the juvenile part of *C. mongolica* shown in **c**; note phosphate biomineralization covered cells with formation of co-marginal ribs. **f** Lateral view of an almost symmetrical sclerite of *C. mongolica*. **g** Apical view of **f**. **h** Lateral view of an asymmetrical sclerite of *C. mongolica* and four co-marginal ridges. **i** Oblique view of an almost symmetrical sclerite of *C. mongolica* with a central fold (“sella”). All images are electron microscope micrographs. IUMP initial primarily unbiomineralized part of the early postembryonic developmental stage of *Camenella*. Specimen identifiers: **a**, **c**, **e** Sal 133-023; **b**, **d** Sal 134-05; **f**, **g** Sal 121-10; **h** Sal 133-10; **i** Sal 133-24.

Co-occurring with eggs, late-stage embryos of *Markuelia* (Fig. 2) and disarticulated SSFs we recovered ten concavo–convex, ovoid disc-like fossils (Figs. 3 and 4) which range in size from 200 × 300 µm to 500 × 700 µm (Supplementary Fig. 1), consistently exhibiting six shallow, approximately bilaterally symmetrical, erect to fully imbricated ridges (Figs. 3a, f, h and 4b, c); we interpret the ridged surface as dorsal, the imbrication of the ridges to reflect anterior–posterior polarity, and size variation to correlate positively with developmental polarity. There is some variation in preservation but the dorsal surfaces of all specimens exhibit an original micromorphology composed of small (7–12 µm diameter) domed polygonal facets (Fig. 3a, b, f, i, 4b, g, f, and 5d). Tomographic data reveal that preservation extends internally (Fig. 4k), with a distinct ventral surface and intervening walls associated with the polygonal borders on the dorsal surface, defining a single polarized layer of cuboidal cellular units. We interpret this as an epithelial cell layer (Fig. 4i–k). None of the specimens preserve additional outer layers covering the cell layer, such as a cuticle or fertilization membrane. The ridges are a development of this dorsal epithelial layer, each ridge V-shaped with an apical spinose projection that is directed posterodorsally. The ridges increase in prominence in correlation with specimen size, reflecting ontogenetic development but also degrees of taphonomic deflation of the ridges (contrast Fig. 3a–h versus Fig. 5b). With only a small number of specimens at hand, it is not yet possible to reconstruct a developmental series. Most preserved individuals represent a similar early developmental stage at a similar size (Supplementary Fig. 1), where the six central V-shaped ridges are still shallow (Figs. 3 and 4a–g). At a later stage, these ridges are more distinct and erect (Figs. 4h and 5b). When erect, the medial ridges have a concave anterior and convex posterior surface (Fig. 5b), facilitating the imbrication seen in other specimens (Figs. 3a–i and 4a–h). At the anterior pole, a small anterior spinose projection occurs in the smallest specimens, appearing larger in concomitantly larger specimens (Figs. 3a, f and 4b, e, h), and as part of a distinct ridge in one specimen (Fig. 3e, h, i). At the posterior pole, a small distinct ridge occurs in the lee of the sixth major medial ridge (e.g., Fig. 3c–e).

Six pairs of smaller, posteriorly oriented conical protuberances occur at the periphery of the medial ridges in some better preserved individuals. In the smallest specimens, the peripheral protuberances are clustered ventrally, forming a band-like array of distinct fields (Fig. 4d); the ventral dome-shaped concavity is not exposed. In larger specimens, the ventral surface is otherwise dominated by a concavity elongated along the anterior–posterior axis, with a limiting circumferential ridge which extends ventral of the paired peripheral ridges (Fig. 3c), tapering to the position of the anterior spine. Together, these features comprise a total of 18 bilaterally arranged structures; six medial ridges and six pairs of marginal conical protuberances, all of which we consider as nascent sclerites.

## Discussion

### Affinity of the early postembryonic bilaterian developmental stages

The polarized bilateral symmetry and dorsoventrally differentiated organization of these demonstrably multicellular fossil organisms is compatible only with a total-group bilaterian animal affinity. The exposed cellular epithelial organization of the dorsal surface is strongly reminiscent of embryonic stages of animal development, in which a protective integument has yet to develop. However, the size range of the developmental stages is indicative of growth, which only occurs in post-hatching stages (Supplementary Fig. 1). Moreover, while associated eggs and embryos of *Markuelia* are mostly preserved with fertilization envelopes, the new bilaterian fossils are not, indicating that they represent postembryonic developmental stages. The fossils are not completely preserved; there must have been more to the soft-tissue anatomy of this organism than the epithelial layer comprising the dorsum. The ventral concavity must represent the location of the main corpus of the organism. There is little direct evidence to constrain ventral anatomy, but body orifices, locomotory, and sensory organs must have been directed ventrally since, given the cellular fidelity of preservation, they would be observable if present dorsally. This anatomical interpretation is compatible with a slug-like bodyplan comparable to those of the acuiliferan molluscs, including crown-polyplacophorans, stem-polyplacophorans such as *Echinochiton*^31^, and stem-acuiliferans such as *Halkieria*^32^ and *Calvapilosa*^33^. Indeed, the morphology of the central ridges is reminiscent of the early development of sclerites in modern chitons^34^, where distensions in the soft tissue of the trochophora larva and the earliest hatchlings represent the precursor of biomineralized plates in the juveniles. However, the overall morphology is also compatible with stem-molluscs such as *Wiwaxia*^35^, as well as more enigmatic taxa such as *Orthozanclus*^36^ and *Paracarinachites*^37^, and it is the inferred bodyplan of putative early stem-brachiopods, such as the camenellan tommotiids^16–23,36,38,39^.

Most of these comparisons are very general and amount to little more than the vicarious slug-like gestalt. These juveniles clearly lack the shell plates of stem- and crown-acuiliferans, which is presumably a juvenile feature of sachitids. They also exhibit a simpler organization than the complex sclerotome of sachitids, *Orthozanclus* and *Wiwaxia*. *Paracarinachites* is also difficult to rationalize with the anatomy of the new fossils, not least since its serial organization reflects marginal sequential addition to the scleritome, not an initially metameric organization. Furthermore, we did not recover remains of these groups in our samples, some of which are known only from much older^37^ and younger^31^ strata. However, the morphology of the broad-based medial ridges and their apical spines in the unmineralized juvenile is particularly reminiscent of the mineralized sclerites of co-occurring sclerites attributed to *Camenella* (Fig. 5a, c, f–i). Furthermore, the isolated sclerites of *Camenella* in this assemblage preserve at their tips a pattern of epithelial cell imprints comparable to the dorsal epithelium of these juvenile stages (Fig. 5a, c, e). We therefore propose that these bilaterian fossils represent early postembryonic developmental stages of co-occurring camenellans, and that the ridges and conical protuberances on their dorsal surface reflect an early nonskeletal stage in the morphogenesis of camenellan sclerites which developed from this epithelium. We consider these early postembryonic developmental stages because of the morphological and structural changes required in establishing the adult bauplan of camenellans. These include further differentiation into three discrete types of sclerites, the development of co-marginal ribbing on the sclerites and, in particular, the onset of biomineralization.

**Fig. 6:**
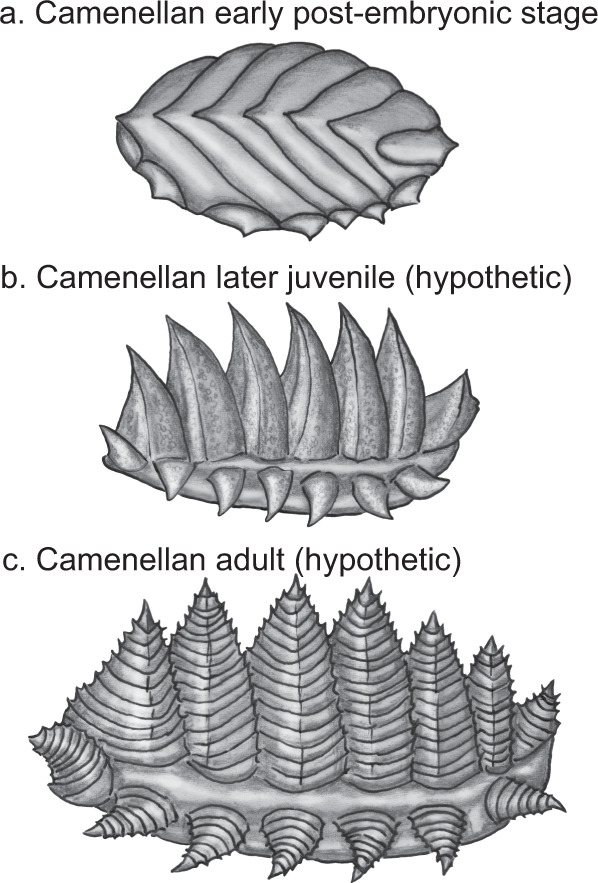
Reconstruction of dorsal morphology in camenellans. **a** Interpretative reconstruction of camenellan early postembryonic developmental stage with six central-arched shallow ridges and six pairs of strongly asymmetric lateral protuberances of peripheral zone. **b** Hypothetical reconstruction of dorsal scleritome in juvenile camenellan before onset of biomineralization in sclerites. **c** Hypothetical reconstruction of dorsal scleritome in an adult camenellan with biomineralized sclerites organized in a medial and a peripheral concentric zone.

The number of ridges and conical protuberances in the dorsal surface of the bilaterian postembryonic developmental stages suggests that early in its development, the scleritome was composed of 20 sclerites arranged in two zones, a medial zone with more symmetrical sclerites (albeit often with slight asymmetry or torsion in mineralized sclerites of adults) and a peripheral zone with strongly asymmetric sclerites (Fig. 6). As such, we recognize three different types of sclerites in the scleritome instead of two interpreted by Bengtson^16^ and Skovsted et al.^38^ for *Camenella*: (a) six large saddle-shaped central sclerites (Fig. 5f, g), (b) one broader and spinose anterior sclerite and a small posterior sclerite (Fig. 5i), as well as (c) six pairs of strongly asymmetric lateral sclerites (Fig. 5h). The three sclerite morphotypes have already been recognized in the kennardiid *Dailyatia*^39^ and are likely to occur also in kelanellids, such as *Kelanella* and *Lugoviella*.

Camenellans have been known hitherto only from disarticulated sclerites and the nature of their bodyplan and that of other tommotiids, has been the subject of protracted conjecture^16–23,38,39^. They are implicated in debates over the nature of tommotiid bodyplans, the evolutionary assembly of the brachiopod bodyplan, and (almost ironically) utilized as an interpretative model for resolving the affinity of halkieriids. Following Bengtson^16^, the bodyplan of camenellans has invariably been inferred as slug-like, with a multimembrate dorsal scleritome. Bengtson^16^ reconstructed the scleritome as composed of serial transverse rows comprised of a single medial approximately symmetrical mitral sclerite flanked by a pair of asymmetric sellate sclerites. This model was followed in reconstructing the scleritomes of other camanellans (e.g., refs. ^38,39^) and adapted for other tommotiids^16–23^. Based solely on isolated sclerites, it is astonishing that these reconstructed scleritomes bear any resemblance to reality, let alone the very precise correspondence of the bilaterian postembryonic developmental stages to the original reconstruction of Bengtson^16^. Based on the consistent occurrence of ridges and protuberances in the juveniles, we hypothesize that adult camenellans developed a dorsal scleritome with organophosphatic assymetric sclerites arranged in the peripheral zone of a slug-like body and more symmetrical sclerites in the central medial zone, and with some morphological variation in the anterior position (Fig. 6).

The bilaterian postembryonic developmental stages from the Salanygol Formation suggest that tommotiids manifest diverse bodyplans, adding the slug-like bauplan of camenellans to the tubular bodyplan of eccentrothecan tommotiids^40^, and the bivalved bodyplan inferred for *Micrina*^41^. Camenellans are commonly accepted as early diverging members of the brachiopod stem lineage^36,38,42^.

**Fig. 7:**
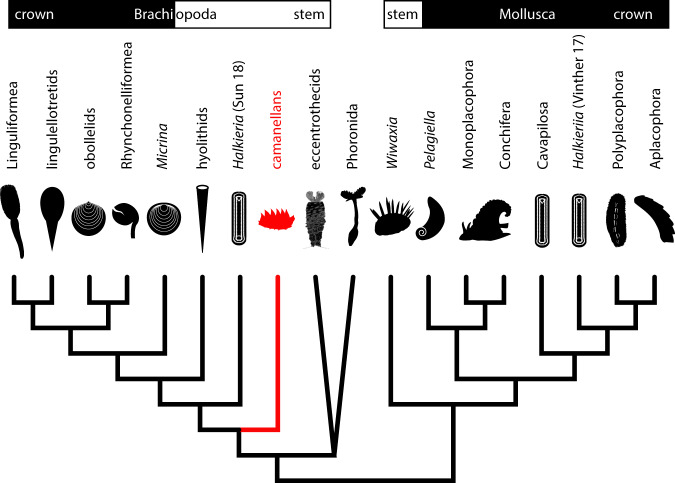
Phylogenetic position of camenellans and other tommotiid clades among crown-brachiopods, -phoronids, -molluscs, and their respective stem lineages (modified from refs. ^36,42^). The common ancestor of the molluscan and brachiopod stem-lineages is likely reconstructed as a slug-like vermiform organism with multiple sclerites arranged in concentric zones. The differing phylogenetic positions for *Halkieria* reflect the contrasting interpretations Vinther et al.^33^ and Sun et al.^42^, while the phylogenetic position of eccentrothecids and phoronids are presented as an unresolved consensus of recent studies^40,42,45^.

Camenellans developed a complex dorsal scleritome organized into a medial and peripheral zone (Fig. 6), similar to sachitids with three concentric zones and polyplacophorans with two concentric zones. Indeed, Zhao et al.^36^ rejected a sachitid affinity for *Halkieria*^33^ allying it and *Orthozanclus* to the camenellans (compare Fig. 7). To them and others^43^, this similarity reflects brachiopods and molluscs having descended from a slug-like common ancestor with a dorsal, serially organized scleritome that may or may not have been biomineralized. They consider but dismiss the challenges of this hypothesis, including the conflicting mineralogies of sachitid and tommotiid sclerites. However, the constructional morphology of camenellan sclerites, marshaled in support of a brachiopod affinity^38^, is not readily compatible with the sclerites of halkieriids, which are more comparable to chiton sclerites^44^. Together, this suggests that though mineralized sclerites may not be a shared primitive feature of a brachiopod plus mollusc clade, a vermiform slug-like bodyplan may well reflect the nature of the common ancestor of these trochozoan phyla (Fig. 7). This conclusion is incompatible with the interpretation of the tubiculous eccentrothecids and phoronids as an outgroup to brachiopods and tommotiids, including camenellans, which has been used to support a tubiculous origin of the bivalved brachiopod bodyplan^40,45^. However, the phylogenetic position of eccentrothecids and phoronids, relative to one another, as well as to other trochozoans, remains highly contentious, with most phylogenetic schemes resolving them as comprising a clade^40,45^, and as closer relatives of crown-brachiopods than camenellans^36,45^. As such, the tubiculous bodyplan of eccentrothecids and phoronids is derived from a slug-like bodyplan. It is worth noting that *Camenella* and other tommotiid genera are most common in the Cambrian Stage 3, where they co-occur with the oldest crown-group brachiopods, suggesting some evolutionary prehistory to the brachiopod total group.

### An unusual fidelity of preservation in the new Salanygol lagerstätte

Most previous reports of phosphatized invertebrate eggs, embryos and juveniles, and larvae have been documented from cratons with Ediacaran–Cambrian cover strata containing large phosphate deposits, such as the Yangtze Platform^7,10–12,14^ and Australia^15,46^. Reports from strata unaccompanied by sedimentary phosphate deposits, such as Siberia^4^ and Laurentia^15^, are rarer still and largely preserve only embryonic and postembryonic developmental stages in which cuticle develops precociously. This new report from the Cambrian Stage 3 Salanygol Formation of Mongolia is significant in that it is unaccompanied by sedimentary phosphate deposits and yet, alongside the cuticular embryos of *Markuelia*, embryos encased in extracellular fertilization membrane and articulated cuticularized worm-like organisms (Fig. 2) it preserves uncuticularised early postembryonic developmental stages with a cellular level of fidelity not seen previously outside of sedimentary phosphate deposits. The early Ediacaran Weng’an Biota and the early Cambrian Kuanchuanpu Formation, both on the Yangtze Platform, preserve embryos to a cellular and subcellular level^47–49^, but they do not preserve postembryonic stages with cellular fidelity. This fine scale of preservation is commonly referred to Orsten-style preservation where soft-bodied remains are replicated through impregnation or templating with calcium phosphate^50^. However, Orsten-style preservation is almost exclusively limited to the preservation of recalcitrant cuticle and gut contents; cellular scale preservation is almost unknown^50^. In this sense, the cellular scale preservation of embryos is quite distinct, attributed to the geochemical microenvironments created by enclosure within a fertilization envelope that inhibits decay and promotes authigenic mineralization^51^. Experimental evidence has suggested that the preservation of uncuticularised juvenile stages^52^ is not possible, since they decay rapidly and even when microbial degradation is suppressed, the constituent cells lose cell adhesion and are dispersed^51^. The cellularly preserved early postembryonic developmental stages from the Salanygol Formation demonstrate that these experimental insights are not limiting on the fossil record more generally. They prompt the need for a better experimental understanding of the mechanisms by which cellular scale preservation of developmental stages can be fossilized. The Salanygol assemblage also raises the potential for discovering a greater breadth of life developmental stages from the fossil record, and expands the scope of facies and stratigraphic intervals, in which they may be discovered.

The soft-bodied fauna from the Salanygol Formation preserves an assemblage of phosphatized eggs and late-stage embryos of *Markuelia* alongside early postembryonic development stages of a slug-like metameric bilaterian. We interpret the bilaterian developmental stages as juveniles of camenellans represented in the same fossil deposit by adult sclerites. As such, the camenellan juveniles appear to corroborate the widely held, but unsubstantiated view that these camenellan tommotiids had a slug-like bodyplan with a dorsal scleritome, perhaps reflecting a slug-like bodyplan for ancestral trochozoans. It is likely that a broader diversity of developmental stages and taxa are preserved within the fossil lagerstätte, whose characteristics raise great hope for comparable discoveries in similar facies from other geographic regions and stratigraphic intervals. These will serve to flesh out our understanding of the embryology of early animals and the emergence of the bodyplans that underpin extant animal biodiversity.

## Methods

The phosphatized embryos, postembryonic developmental stages, and adult sclerites were extracted with 10% buffered acetic acid from carbonates, followed by sieving (>64 and >125 µm) and hand-picking of the acid-resistant residue (fraction >125 µm <2 mm) under a binocular microscope. Scanning electron microscopy (SEM) was carried out at the Department of Earth Sciences, Freie Universität Berlin with a ZEISS Supra 40 VP Ultra (Carl Zeiss Microscopy GmbH, Oberkochen, Germany). Specimens selected for investigation in SEM were rinsed in pure ethanol, mounted on aluminum sample stubs with adhesive carbon film, and sputtered for 6 min with gold in a BioRad elemental SEM coating system. Synchrotron Radiation X-ray Tomographic Microscopy (srXTM) was conducted at the X02DA TOMCAT beamline of the Swiss Light Source, Paul Scherrer Institut, Villigen, Switzerland. Measurements were obtained with an operating voltage of 15 KeV, exposure of 150 ms, using a 20 µm LuAg:Ce scintillator, and a 20× objective, yielding reconstructed tomographic data with voxel dimensions of 0.325 µm. A total of 1501 projections were taken equiangularly through 180^o^ of rotation within the beam. Projections were post-processed and rearranged into flat- and dark-field-corrected sinograms, and reconstruction was performed on a 60-core Linux PC farm, using a highly optimized routine based on the Fourier transform method and a regridding procedure^53^. Slice data were analyzed and manipulated using VGStudioMax 2.0 (www.volumegraphics.com). Figures were assembled using the computer software Adobe Photoshop CS6, Adobe Illustrator CS6 and Microsoft Ecxel 14.2 for Mac, while texts were handled by Microsoft Word 14.2 for Mac.

### Reporting summary

Further information on research design is available in the Nature Research Reporting Summary linked to this article.

## Supplementary information

Supplementary Information

Reporting Summary

## Data Availability

Tomographic data are freely available from the University of Bristol Research Data Depository at https://data.bris.ac.uk/data/dataset/27qu5twt57gu62m9dpk4ntcrfs, 10.5523/bris.27qu5twt57gu62m9dpk4ntcrfs. All figured specimens are housed at the Department of Earth Sciences, Freie Universität Berlin (FUB).
