# Supplementary material for: Exceptionally preserved early Cambrian bilaterian developmental stages from Mongolia
Source: Nat Commun. 2021 Feb 15;12:1037. doi: 10.1038/s41467-021-21264-7 (PMC7884407; doi:10.1038/s41467-021-21264-7)
Supplement: Supplementary file 1 — Supplementary Information [file 41467_2021_21264_MOESM1_ESM.pdf]

Supplementary Information

Exceptionally preserved early Cambrian bilaterian developmental stages from Mongolia

Steiner et al.

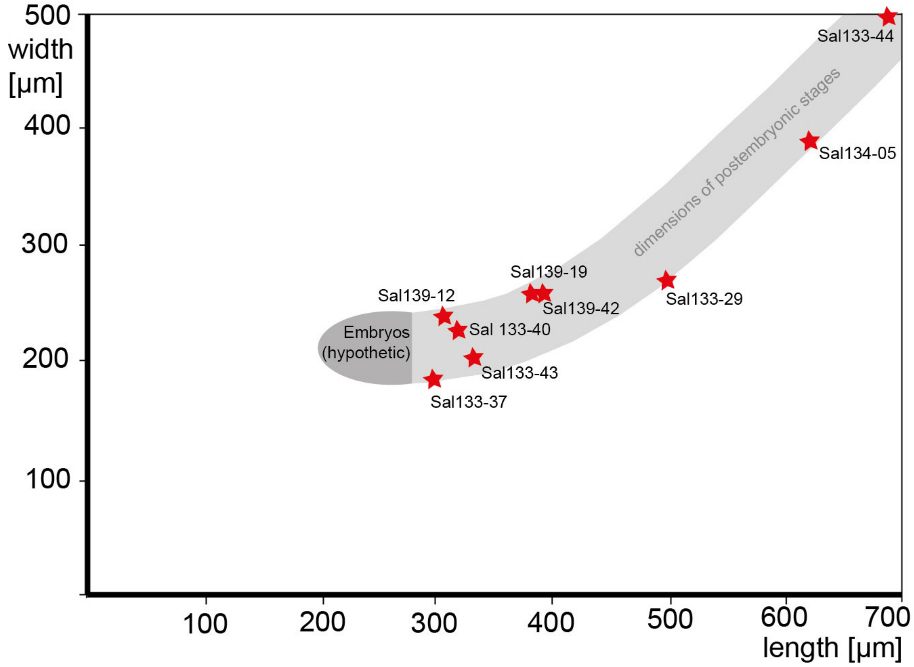

Supplementary Figure 1. Size range of studied camenellan juveniles. Most individuals represent a similar early developmental stage. Later developmental stages are represented by two individuals (Sal133-44; Sal134-005) with more erect nascent sclerites of which sample Sal134-005 may partly be incomplete.
