# Supplementary material for: Exceptionally preserved early Cambrian bilaterian developmental stages from Mongolia
Source: Nat Commun. 2021 Feb 15;12:1037. doi: 10.1038/s41467-021-21264-7 (PMC7884407; doi:10.1038/s41467-021-21264-7)
Supplement: Supplementary file 2 — Reporting Summary [file 41467_2021_21264_MOESM2_ESM.pdf]

nature research

Corresponding author(s): Steiner, Michael

Last updated by author(s): Dec 20, 2020

Reporting Summary

Nature Research wishes to improve the reproducibility of the work that we publish. This form provides structure for consistency and transparency in reporting. For further information on Nature Research policies, see Authors & Referees and the Editorial Policy Checklist .

Statistics

For all statistical analyses, confirm that the following items are present in the figure legend, table legend, main text, or Methods section.

n/a	Confirmed	
☒	☐	The exact sample size (*n*) for each experimental group/condition, given as a discrete number and unit of measurement
☒	☐	A statement on whether measurements were taken from distinct samples or whether the same sample was measured repeatedly
☒	☐	The statistical test(s) used AND whether they are one- or two-sided<br>*Only common tests should be described solely by name; describe more complex techniques in the Methods section.*
☒	☐	A description of all covariates tested
☒	☐	A description of any assumptions or corrections, such as tests of normality and adjustment for multiple comparisons
☒	☐	A full description of the statistical parameters including central tendency (e.g. means) or other basic estimates (e.g. regression coefficient) AND variation (e.g. standard deviation) or associated estimates of uncertainty (e.g. confidence intervals)
☒	☐	For null hypothesis testing, the test statistic (e.g. *F*, *t*, *r*) with confidence intervals, effect sizes, degrees of freedom and *P* value noted<br>*Give P values as exact values whenever suitable.*
☒	☐	For Bayesian analysis, information on the choice of priors and Markov chain Monte Carlo settings
☒	☐	For hierarchical and complex designs, identification of the appropriate level for tests and full reporting of outcomes
☒	☐	Estimates of effect sizes (e.g. Cohen's *d*, Pearson's *r*), indicating how they were calculated

Our web collection on statistics for biologists contains articles on many of the points above.

Software and code

Policy information about availability of computer code

Data collection	Not applied
Data analysis	Word 14.2 for Mac; Excel 14.2 for Mac; Adobe Photoshop CS6; Adobe Illustrator CS6; VG StudioMax2.0

For manuscripts utilizing custom algorithms or software that are central to the research but not yet described in published literature, software must be made available to editors/reviewers. We strongly encourage code deposition in a community repository (e.g. GitHub). See the Nature Research guidelines for submitting code & software for further information.

Data

Policy information about availability of data

All manuscripts must include a data availability statement. This statement should provide the following information, where applicable:

- Accession codes, unique identifiers, or web links for publicly available datasets
- A list of figures that have associated raw data
- A description of any restrictions on data availability

Tomographic data are freely available from the University of Bristol Research Data Depository at https://data.bris.ac.uk/data/dataset/27qu5twt57gu62m9dpk4ntcrfs; doi <10.5523/bris.27qu5twt57gu62m9dpk4ntcrfs>.

Field-specific reporting

Please select the one below that is the best fit for your research. If you are not sure, read the appropriate sections before making your selection.

☐ Life sciences ☐ Behavioural & social sciences ☒ Ecological, evolutionary & environmental sciences

Ecological, evolutionary & environmental sciences study design

All studies must disclose on these points even when the disclosure is negative.

Study description	We describe and interprete a set of fossilized Cambrian invertebrate eggs, embryos, larvae, and juveniles which have hitherto not been described. For the systematic size measurements of specimens all individuals were measured and investigated.
Research sample	We describe fossilized Cambrian camenellan juveniles and co-ocurring remains of the Tommotiid Camenella mongolica, which is interpreted as a stem-group representative of brachiopod lineage
Sampling strategy	For microfossil extraction we sampled carbonatic sediments in regular distances (50 cm oder lower stratigraphical distance); alltogether 50 samples were collected and all processed and investigated for fossilized fauna; only 3 samples (nos. Sal 133, 134, 139) contained the new eggs, embryos and postembryonic stages
Data collection	The stratigraphical sediment sequence in Mongolia was measured and documented by Steiner & Yang Ben, partly supported by Hohl and Li Da. All sediment samples were cut by a diamond saw in the lab of FU Berlin, one small slab of each sediment sample was stored as a reference at FU Berlin, Dpt. Geosciences; all sediment samples were chemically processed for microfossil extraction as described in the article; all sample residues were hand-picked by Steiner and all embryos and postembryonic stages were systematically studied under SEM at FU Berlin; synchrotron radiation X-ray Tomographic Microscopy (srXTM) was conducted at the X02DA TOMCAT beamline of the Swiss Light Source, Paul Scherrer Institut, Villigen, Switzerland by Phil Donoghue and the tomographic processing of data done by Michael Steiner
Timing and spatial scale	Samples were collected during fieldwork between July, 14, 2017 and July, 17, 2017; resampling of same locality for a test of reproducibility was carried out in June, 19-20 , 2019;<br>samples of original sampling were processed in 2018 and final investigation and imaging at SEM were done until September 2019
Data exclusions	no data relevant to the study of the new occurrence of fossilized invertebrate eggs, embryos and postembryonic stages were excluded
Reproducibility	the stratigraphical horizons yielding fossilized embryos and juveniles were re-sampled in June 2019 and these samples proved reproducibility for the fossils
Randomization	n/a
Blinding	blinding is not relevant to a palaeontological study

Did the study involve field work? ☒ Yes ☐ No

Field work, collection and transport

Field conditions	Sampling was carried out in the Khasagt-Khairkhan Mountain range, under conditions of continental dry climate; however, this has not a strong relevance to the study of fossil organisms, except for the weathering conditions of the sediment strata containing the fossil association; the fossiliferous strata showed moderate weathering impact, since the samples are taken from natural rock exposures of a Mountain range
Location	Samples are taken from the Salanyi Gorge (GPS N46°48´32.1” E095°46´18.8”) of western Mongolia; Altitude ca.2400 m
Access and import/export	Scientific cooperation with Mongolian partners was based on an agreement, signed by the head of Geological Research Center, Prof. Ochir Gerel on May, 17th, 2016. Permits to study the mountain range were applied and obtained by our Mongolian cooperation partner Anaad Chimedseren at Mongolian University of Science and Technology, Ulan Bator;<br>Export licences and customs clearance for the systematic study of samples at FU Berlin, Germany were applied and obtained by our Mongolian cooperation partner Anaad Chimedseren at Mongolian University of Science and Technology, Ulan Bator. Samples were inspected by the Mongolian authorities prior to export, such as the Geology Agency of Mongolia.<br>Rock sample collection was carried out in natural rock exposures. Influence on natural habitates during rock collection and in camp sites were kept at a minimum.
Disturbance	Samples were taken by crack out of a small amount of rock from a natural rock exposure with geological hammers; we tried to keep our work impact on natural soils and fauna and flora at a minimum. All waste produced during the camp operation was carried back from the natural habitate to Altai city.

Reporting for specific materials, systems and methods

We require information from authors about some types of materials, experimental systems and methods used in many studies. Here, indicate whether each material, system or method listed is relevant to your study. If you are not sure if a list item applies to your research, read the appropriate section before selecting a response.

Materials & experimental systems

n/a	Involved in the study
☒	☐ Antibodies
☒	☐ Eukaryotic cell lines
☐	☒ Palaeontology
☒	☐ Animals and other organisms
☒	☐ Human research participants
☒	☐ Clinical data

Methods

n/a	Involved in the study
☒	☐ ChIP-seq
☒	☐ Flow cytometry
☒	☐ MRI-based neuroimaging

Palaeontology

Specimen provenance

Specimens are collected from early Cambrian limestones of Salanyigol Formation at Salanyi Gorge, Altai Region, Mongolia.

Specimen deposition

The specimens are curated in the Research Collection at Department of Earth Sciences, Freie Universität Berlin, Germany and accessible via contact to the senior author

Dating methods

no new age datings were carried out in our study; the ages of the samples are derived from relative age datings of stratum by co-ocurring macro-fauna (Kruse et al. 1996, Sosnovskaja et al. 2019) as cited in our article and by chemostratigraphy (Smith et al. 2016)

☒ Tick this box to confirm that the raw and calibrated dates are available in the paper or in Supplementary Information.
